# Adsorption of Organic Compounds on Adsorbents Obtained with the Use of Microwave Heating

**DOI:** 10.3390/ma15165664

**Published:** 2022-08-17

**Authors:** Aleksandra Bazan-Wozniak, Judyta Cielecka-Piontek, Agnieszka Nosal-Wiercińska, Robert Pietrzak

**Affiliations:** 1 Faculty of Chemistry, Adam Mickiewicz University in Poznań, Uniwersytetu Poznańskiego 8, 61-614 Poznań, Poland; 2 Department of Pharmacognosy, Poznan University of Medical Sciences, Rokietnicka 3, 60-806 Poznan, Poland; 3 Faculty of Chemistry, Maria Curie-Sklodowska University in Lublin, Maria Curie-Sklodowska 3, 20-031 Lublin, Poland

**Keywords:** activated carbon, microwave heating, cationic and anionic dyes, kinetic and equilibrium study, thermodynamic study

## Abstract

Activated carbons were obtained by physical and chemical activation of the residue of supercritical extraction of green tea leaves. All the adsorbents obtained were characterized by: elemental analysis, low-temperature nitrogen adsorption, and the contents of acidic and basic oxygen functional groups on the surface of activated carbons by the Boehm method. The activated carbons were micro- or micro-mesoporous with well-developed surface area ranging from 520 to 1085 m^2^/g and total pore volume from 0.62 to 0.64 cm^3^/g. The physical activation of the precursor led to the strongly basic character of the surface. Chemical activation with 50% solution of H_3_PO_4_ of the residue of supercritical extraction of green tea leaves promoted the generation of acidic functional groups. All adsorbents were used for methylene blue and methyl red adsorption from the liquid phase. The influence of the activation method, pH of the dye solution, contact time of adsorbent with adsorbate, the temperature of adsorption, and rate of sample agitation on the effectiveness of organic dyes removal was evaluated and optimized. In the process of methylene blue adsorption on adsorbents, an increase in the sorption capacity was observed with increasing pH of the adsorbate, while in the process of methyl red adsorption, the relation was quite the reverse. The adsorption data were analyzed assuming the Langmuir or Freundlich isotherm models. The Langmuir model better described the experimental results, and the maximum sorption capacity calculated for this model varied from 144.93 to 250.00 mg/g. The results of the kinetic analysis showed that the adsorption of organic dyes on activated carbon was following the pseudo-second-order model. The negative values of the Gibbs free energy indicate the spontaneous character of the process.

## 1. Introduction

When taking into account the high biotoxic effect of sewage [1], both municipal and industrial, it should be subjected to treatment, including purification, and one of the processes used for this purpose is adsorption [2,3]. The widespread use of this method of purification entails increasing demand for efficient adsorbents [4,5,6].

One of the most popular adsorbents used for the removal of liquid pollutants of either organic or inorganic origin is activated carbon [7,8]. Materials based on activated carbon show excellent adsorption properties determined by the highly-developed porous structure and high surface reactivity [9,10,11,12]. A large variety of materials with high carbon content (hard (bituminous) coal, brown coal, coke, peat, and wood) from which activated carbon adsorbents can be obtained translate into a low price and a wide spectrum of applications [13,14,15]. Interestingly, activated carbon-based adsorbents can be obtained from the waste products of some technological processes, e.g., plant-origin products that are residues of plant supercritical extraction [16,17].

The properties of activated carbon-based adsorbents depend on the type of source of carbon used, so the first step in the search for the right adsorbent is a careful choice of the raw material, the method of activation, activating factor, and conditions of carbonization and activation [18,19].

Another factor influencing the physical and chemical properties of activated carbon is the method of heating [20]. In the production of activated carbon adsorbents, the heating stage is realized by the conventional method or with the use of microwaves [21]. Microwave heating is homogeneous, selective, and fast; moreover, it permits immediate control of the process. The heating takes place in the whole volume of the sample, so the final temperature is reached faster, which reduces the energy and time of the process, so also the cost of production of activated carbon [22]. According to the literature data, the use of microwave heating leads to a considerable reduction in electric energy consumption, so the energy cost and detailed calculations have been made by Ao et al. [23]. Moreover, the authors of [24] have calculated that the cost of production of 1 kg of activated carbon obtained from the sludge of a tannery-treatment effluent plant with the use of microwave heating was USD 32.43, while the production of 1 kg of activated carbon with the use of conventional heating was USD 70.67. These numbers illustrate the scale of cost reduction when using microwave irradiation as a source of heating. In order to obtain activated carbons of well-developed specific surface area and microporous structure, single-stage chemical activation is applied. The choice of an appropriate activating agent is important. One of the attractive activating agents is phosphoric acid [25], which can be easily recovered by washing with water; moreover, its toxicity and harmful effect on the environment are lower than those of zinc chloride [26]. The use of alkali hydroxides as activating agents is expensive and hazardous, apart from the fact that these compounds show corrosive properties [27]. Another advantage of activation with H_3_PO_4_ is the possibility of using lower temperatures than in the processes with other activating agents. The process of physical activation involves two subprocesses: pyrolysis of the precursor followed by its activation [28]. Physicochemical processes of the pyrolysis products depend on the type of precursor and parameters of the process, i.e., the rate of temperature increase, final temperature, atmosphere, pressure, and duration. Unfortunately, even with the carefully chosen parameters of the first step of physical activation (carbonization), it leads to activated carbon adsorbents of relatively low adsorption capacity. Therefore, an additional activation process (the second step of physical activation) is performed to unlock the pores and develop the porous structure, so to improve the adsorption properties [29,30].

This paper reports the obtaining of physically and chemically activated carbon adsorbents from the residue of supercritical extraction of green tea leaves and their characterization. The process of supercritical extraction fits in the assumptions of ‘green chemistry’; however, the wide range of applications of this process implies the generation of considerable amounts of waste that must be dealt with. Literature does not provide much information on the preparation of carbon materials from post-critical extraction waste, which has been a direct inspiration for undertaking this study. The novelty of our approach is the use of the residue of supercritical extraction of green tea leaves as a precursor for the synthesis of carbon-based adsorbents.

The adsorption capacities of the obtained activated carbon adsorbents towards the model organic compounds (methylene blue, methyl red) were assessed by UV-Vis spectrophotometric method. In order to explain the mechanism of adsorption, the impacts of a number of factors that may affect the process (the activation method, pH of the dye solution, contact time of adsorbent with adsorbate, temperature of adsorption, and rate of sample shaking) were evaluated.

## 2. Materials and Methods

### 2.1. Materials and Chemical Reagents

The precursor of activated carbon adsorbents were residues of supercritical extraction of green tea leaves (P). The residues were washed a few times with distilled water in order to remove ash and pollutants, and then the material was dried to constant mass at 105 °C.

Methyl red and methylene blue as model adsorbates were purchased from Avantor Performance Materials Poland S.A. (Gliwice, Poland). Hydrochloric acid, phosphoric(V) acid, and sodium hydroxide were purchased from Merck (Darmstadt, Germany). The solutions needed were made with deionized water.

### 2.2. Preparation of Adsorbents

The amount of precursor to be used was divided into two parts. One of them was mixed with a 50% solution of H_3_PO_4_ at the weight ratio of 1:2. The impregnated sample was then subjected to thermochemical treatment in a microwave oven (Phoenix, CEM Corporation, Matthews, IL, USA) at 500 °C for 30 min. The sample was heated at a rate of 5 °C/min in a neutral gas atmosphere (nitrogen—330 mL/min) and labeled as AC.

The other part of the precursor was subjected to carbonization at 300 °C (in nitrogen flow of 170 mL/min) for 60 min and then to activation by CO_2_ (flow rate of 250 mL/min) at 600 °C for 30 min. The process of physical activation was performed in the same microwave oven as used for chemical activation described above. After the activation, the sample was cooled in nitrogen atmosphere. This sample was labeled as AF.

The prepared activated carbon samples: AC was washed with hot distilled water, and AF was washed with hydrochloric acid and hot distilled water until the filtrate was of neutral pH. Then the samples were dried at 105 °C to constant mass.

### 2.3. Instrumentation

Elemental analyses of the precursor and activated carbons were performed with the use of Vario ELIII elementalanalyzer (ElementarAnalysensysteme GmbH, Langenselbold, Germany). The textural parameters of the obtained adsorbents were characterized on the basis of the low-temperature nitrogen adsorption/desorption, using AutosorbiQ, provided by Quantachrome Instruments (Boynton Beach, FL, USA). The acid-base properties of the samples were assessed following the procedure described in [31].

The sorption capacities of the activated carbon samples were assessed using a spectrophotometer Carry 100 Bio (Agilent, SantaClara, CA, USA). The solutions of the dyes studied with the activated carbon samples were stirred on a laboratory shaker (Heidolph, Schwabach, Germany). All studies were performed using a laboratory centrifuge Frontiner™ centrifugeFC5515 (OHAUS, Parsippany, NJ, USA), while pH measurements were made with a BlueLine 25 pH electrode (SI Analytics, Weilheim, Germany).

### 2.4. Adsorption Process

#### 2.4.1. Preparation of Solutions

The stock solutions of 1000 mg/L concentration of each dye were prepared. A portion of 1000 mg of each dye was dissolved in deionized water in measuring flasks of 1000 mL in capacity. The solutions of other concentrations (mg/L) to be used in the study were prepared from the stock solutions by dilution.

#### 2.4.2. Dye Concentration

Adsorption of the dyes was performed using the following procedure. Glass bottles of 100 mL in capacity were charged with 20 mg of a given adsorbent and flooded with 50 mL of a dye solution with initial concentrations in the range from 10 to 120 mg/L. The samples were shaken on the shaker for 7 h at 200 rmp/min. Then the samples were centrifuged for 5 min, and absorbance of the filtrates was measured by the UV-Vis spectrometer at the wavelength corresponding to a given dye (Table 1).

The amounts of the adsorbed dye (*q_e_*) was calculated from the Formula (1):

(1) $$q_{e}=\frac{C_{0}-C_{e}}{m}\cdot V$$

where: *C*_0_—initial dye concentration (mg/L); *C_e_*—equilibrium dye concentration (mg/L); *m*—weight of the activated carbon (g); *V*—volume of the solution (L).

The results were interpreted assuming the following four models of adsorption: Freundlich, Langmuir, Temkin, and Dubinin–Radushkevich [32]. The linear form of Freundlich isotherm is given by Formula (2). This model is used for characterization of heterogeneity of the adsorbent surface.

(2) $$logq_{e}=logK_{F}+\frac{1}{n}logC_{e}$$

where: *q_e_*—amount of the adsorbed dye (mg/g); *C_e_*—equilibrium concentration [mg/L]; *n*—the Freundlich constant describing intensity of adsorption; *K_F_*—the Freundlich constant describing adsorption capacity (mg/g(mg/L)^1/n^).

The Langmuir model refers to the adsorption of a single layer on the adsorbent surface. The linear form of this model (3) can be written as:

(3) $$\frac{C_{e}}{q_{e}}=\frac{1}{K_{L}\times q_{max}}+\frac{C_{e}}{q_{max}}$$

where: *K_L_*—the Langmuir equilibrium constant (L/mg).

The Langmuir isotherm is also characterized by the dimensionless constant (*R_L_*) known as the equilibrium parameter (4):

(4) $$R_{L}=\frac{1}{1+K_{L}\times C_{0}}$$

Its value informs about the shape of the Langmuir isotherm: (*R_L_* = 1)—linear; (*R_L_* ≥ 1)—unfavorable; (0 < *R_L_* > 1)—favorable; (*R_L_* = 0)—irreversible.

Another model describing the process of adsorption has been proposed by Temkin (5) and is described by the equation:

(5) $$q_{e}=BlnA_{T}+BlnC_{e}$$

where: *B*—a constant equal to *B* = *RT*/*B_T_*; where *B_T_* is the Temkin constant (J/mol); *R*—gas constant (J/mol × K); *T*—temperature (K); *A_T_*—Temkin isotherm equilibrium binding constant (L/mg).

The last model taken into consideration in this study has been proposed by Dubinin–Radushkevich (6) and is given by the equation:

(6) $$lnq_{e}=lnq_{m}-\beta \varepsilon^{2}$$

where: *q_m_*—the monolayer adsorption in the Dubinin–Radushkevich model (mg/g); *β*—a constant describing the sorption energy (mol^2^/kJ^2^); *ε*—adsorption potential.

On the basis of the value of *β* it is possible to find the free energy of adsorption *E* (kJ/mol), (7):

(7) $$E=\frac{1}{\sqrt{2\beta }}$$

The mean free energy gives information about the type of adsorption, chemical or physical, taking place. If *E* < 8 kJ/mol the mechanism involves physical interactions, if *E* comes from the range 8–16 kJ/mol, the dominant mechanism is based on ion exchange, while *E* > 16 kJ/mol indicates that the molecules undergo diffusion in the process of adsorption.

#### 2.4.3. Adsorption Modeling

In order to check the effect of contact time on the effectiveness of the adsorption of the dyes studied, glass bottles were charged with 20 mg of selected activated carbon flooded with 50 mL of either methylene blue (80 mg/L) or methyl red (50 mg/L). The loaded bottles were shaken on a shaker working at the speed of 200 rmp/min. At selected time intervals (10, 20, 30, 40, 50, 60, 120, 150, 180, 240, and 300 min), the absorbance of the solution was measured. The kinetics of adsorption at the dye/activated carbon interface was approximated by two empirical equations, the Langergen one (8) (pseudo-first-order) and Ho and McKay one (9) (pseudo-second-order) [33,34]:

(8) $$\ln\left(q_{e}-q_{t}\right)=lnq_{e}-\frac{k_{1}t}{2.303}$$

(9) $$\frac{t}{q_{t}}=\frac{1}{k_{2}q_{e}{}^{2}}+\frac{t}{q_{e}}$$

where: *q_t_*—the amount of dye adsorbed in a given time (mg/g); *k*_1_—the adsorption constant in the pseudo-first-order equation (L/min); *k*_2_—the adsorption constant in the pseudo-second-order equation (g/mg × min).

#### 2.4.4. pH

The relation between the sorption properties of the activated carbon samples and pH of the dye solution was examined for pH, ranging from 2 to 12. The pH values of the water solutions of methylene blue and methyl red were changed by addition of 0.1 M of NaOH/HCl solution. The samples AC and AF flooded with dye solution of appropriate concentration and pH were agitated (200 rmp/min) for 300 min. Measurements were performed for 20 mg of the activated carbon and 50 mL of the dye solution of an appropriate concentration (methylene blue—80 mg/L, methyl red—50 mg/L) and pH in the range 2–12.

#### 2.4.5. Thermodynamic Study

Thermodynamic studies were carried out in the temperature variants: at 25, 45 or 65 °C. The glass bottles loaded with the samples were shaken (200 rmp/min) and heated for 300 min. The procedure of sample preparation was the same as that used when studying the effect of pH of the dye solution on the adsorption capacities of the carbon adsorbents. The thermodynamics of the adsorption of the dye on the activated carbon adsorbents was characterized on the basis of the enthalpy, entropy, and Gibbs free energy, calculated from the following Equations (10) and (11):

(10) $$∆G^{0}=-RTlnK_{d}$$

(11) $$lnK_{d}=\frac{∆S^{0}}{R}+\frac{∆H^{0}}{RT}$$

where: Δ*G*—Gibbs free energy (kJ/mol); Δ*S*—entropy (kJ/K × mol); Δ*H*—enthalpy (kJ/mol); *T*—absolute temperature (K); *R*—gas constant (8.3144 J/K × mol); *K_d_*—the equilibrium constant equal to *q_e_*/*C_e_*.

#### 2.4.6. Adsorbent Amount

Analysis glass bottles were loaded with different amounts of the two types of activated carbon adsorbents: 10, 20, and 30 mg, and flooded with 50 mL of methylene blue (80 mg/L) or methyl red (50 mg/L). The charged bottles were shaken at the speed of 200 rmp/min for 300 min; then, spectrophotometric measurements were made.

#### 2.4.7. Agitation Rate

The impact of agitation rate on the sorption capacities of the adsorbents studied was checked for two agitation rate values 100 or 200 rmp/min. The process of agitation at a given rate was carried out for 300 min for the samples containing 20 mg of a given activated carbon and methylene blue in a concentration of 80 mg/L or methyl red in a concentration of 50 mg/L.

## 3. Results and Discussion

### 3.1. Characterization of the Precursor

A good precursor of activated carbon should first of all be characterized by a low content of ash and a high content of elemental carbon. As activated carbon adsorbents are in high demand because they are needed in large amounts for the removal of liquid and gas phase pollutants, their popular precursors are cheap materials, often agricultural waste products [35]. A wide range of materials that can be used as precursors of activated carbons means that they show various characteristics, including different contents of ash, humidity, and elemental composition (Table 2). As follows from the data presented in Table 2, the residue of supercritical extraction of green tea leaves has a relatively high content of elemental carbon.

**Figure 1:**
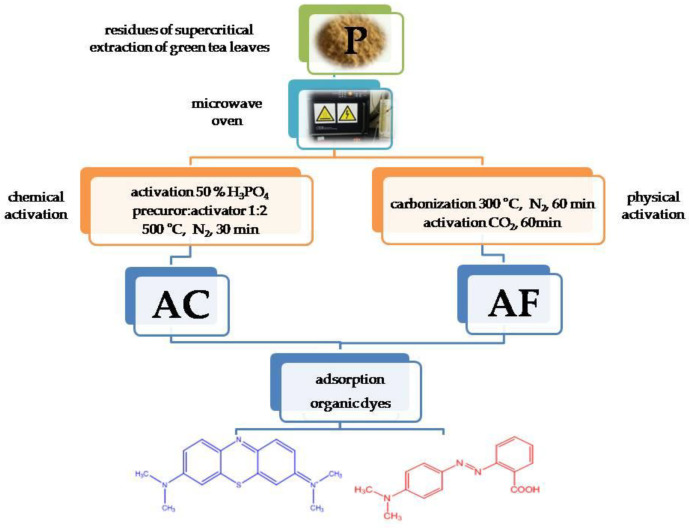
Scheme of the activated carbons preparation.

Only walnut shells [39] have over 10% greater content of elemental carbon than the residue of supercritical extraction of green leaves used in this study. Such a high content of carbon in the precursor (58.8% wt.) is much promising and raises expectations for the high content of this element in the activated carbon adsorbents. Moreover, this precursor showed a low content of ash (2.1% wt.), which is also a desirable characteristic. Two methods of activation were applied: physical and chemical. In order to shorten the time and energy needed for the process, the activated carbon adsorbents were obtained using a microwave oven (Figure 1).

### 3.2. Characterization of Activated Carbon Samples Obtained

The specific surface area, pore volume, and average pore diameter were determined by the BET method. The obtained values of the above parameters are collected in Table 3.

The specific surface area of the carbon adsorbent obtained by chemical activation, sample AC, is 1085 m^2^/g, which is almost twice greater than that of the sample obtained by physical activation (AF) of 520 m^2^/g. The total pore volumes determined for the two samples are similar and fit in the range of 0.62–0.64 cm^3^/g (see Supplementary Figure S1). The different procedures of activation resulted in different textural properties of the two types of activated carbon adsorbents. The sample obtained by chemical activation (50% solution of H_3_PO_4_) shows a distinctly microporous character (1.9 nm), and the average diameter of its micropores is <2 nm. The value of the average pore diameter for the sample obtained by physical activation, AF, is 4.1 nm. The data presented in Table 2 (in particular, the data characterizing sample AC) confirm the advantages following the use of microwave heating (over conventional heating) in the procedure of the adsorbents production. Sample AC (1085 m^2^/g) has better textural properties than the activated carbon obtained with the use of a tube furnace (250 m^2^/g), as described in another scientific paper [17].

As expected, irrespective of the method of activation, the obtained activated carbons showed a much higher content of elemental carbon than the precursor (Table 4).

The highest content of elemental carbon was found in sample AF (88.9% wt.) obtained by physical activation. The higher content of elemental carbon in sample AF may be a consequence of a relatively high activation temperature and a long time of the process, which could stimulate the aromatization of the carbon structure. When using microwave heating, usually, a lower temperature is applied. The increase in the content of elemental carbon resulted in a decrease in the content of hydrogen and nitrogen(H^daf^ 1.4–1.6% wt. and N^daf^ 0.5–0.9% wt.) relative to their content in the precursor (H^daf^ 10.8% wt. and N^daf^ 3.2% wt.; Table 3), while the content of oxygen in samples AC and AF was 14.1 and 10.4% wt., respectively.

**Figure 2:**
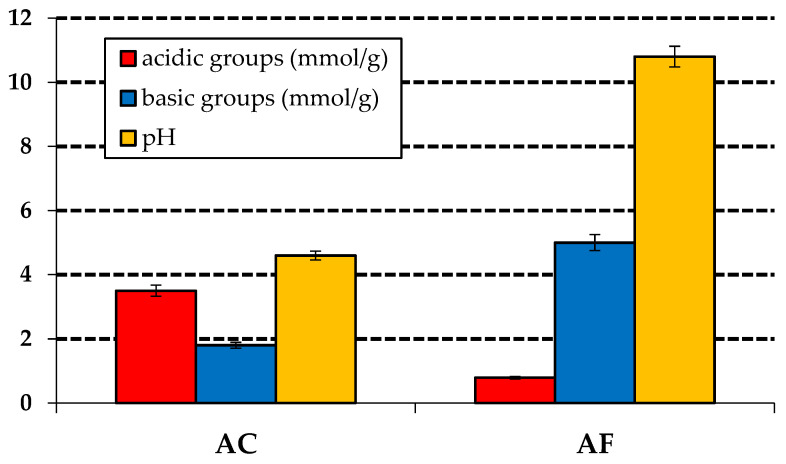
Acid-base properties of the activated carbons.

The content of the mineral substance in the activated carbon adsorbents was determined as 0.3% wt. and 4.3% wt. in samples AC and AF, respectively. The low content of mineral admixtures in these samples follows from the applied washing with hydrochloric acid and distilled water, which is necessary when using the chemical activation of carbon. The use of this washing procedure for the sample obtained by physical activation, AF, confirmed its effectiveness also for the samples subjected to the two-step physical activation process, as the carbon adsorbents obtained by activation with CO_2_ and characterized in our earlier reports had much higher contents of mineral substances [28]. The low content of ash may suggest that the pores in the adsorbents obtained will not be blocked by mineral substances in the process of the adsorption of the dye. The contents of surface oxygen functional groups of acidic and basic character were determined by the method of Boehm titration (Figure 2).

The results indicate that the chemical character of the surfaces of the activated carbon depends on the method of obtaining the carbon adsorbents. The activation of precursor with a 50% solution of H_3_PO_4_ was found to favor the generation of acidic functional groups. The results of the Boehm titration revealed that sample AC had 3.5 mmol/g of acidic functional groups and 1.8 mmol/g of basic ones on the surface, while for sample AF, a much greater predominance of basic functional groups (5 mmol/g) over acidic (0.8 mmol/g) ones was found. The Boehm titration-based conclusions were confirmed by measurements of pH of water extracts of the adsorbents studied, equal to 4.6 and 10.8 for samples AC and AF, respectively.

### 3.3. Adsorption Equilibrium

**Figure 3:**
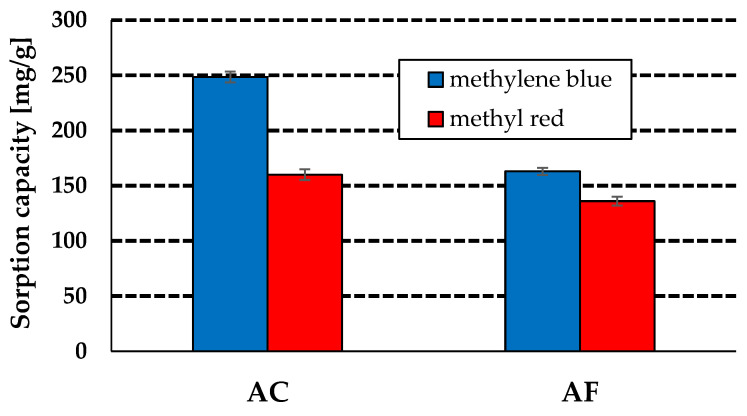
Sorption capacity of activated carbons towards aqueous solutions of methylene blue and methyl red.

The results of sorption capacity measurements towards water solutions of the two organic dyes obtained for the activated carbon samples AC and AF are presented in a bar diagram (Figure 3).

According to these results, the sample obtained by chemical activation proved to be a more effective adsorbent of the dyes. Its sorption capacity towards a water solution of methylene blue is 248 mg/g (initial concentration—120 mg/L), while towards methyl red, it is 163 mg/g (initial concentration—100 mg/L). The sample obtained by physical activation of the residue of supercritical extraction of green tea leaves has an adsorption capacity of 160 mg towards methylene blue and 136 mg towards methyl red. The higher sorption capacity of sample AC is a consequence of a much better developed specific surface area of this sample relative to that of sample AF. A comparison of the data presented in Figure 3 with those published in [17] shows that both samples studied, AC and AF are very attractive against the carbon adsorbents obtained and characterized in [17]. The precursor of the samples presented in the two papers was the residue of supercritical extraction of green tea leaves. The carbon adsorbent characterized in [17] was obtained in a tube furnace by chemical activation with sodium carbonate as the activating agent. It should be emphasized that the sorption capacity of this adsorbent was much lower than the capacities of samples AC and AF, i.e., 85 mg/g and 70 mg/g towards methylene blue and methyl red, respectively. It should also be noted that the production of samples AC and AF was much cheaper than that of the sample characterized in [17]. Moreover, samples AC and AF showed much better textural parameters than the sample studied in the earlier published paper.

**Figure 4:**
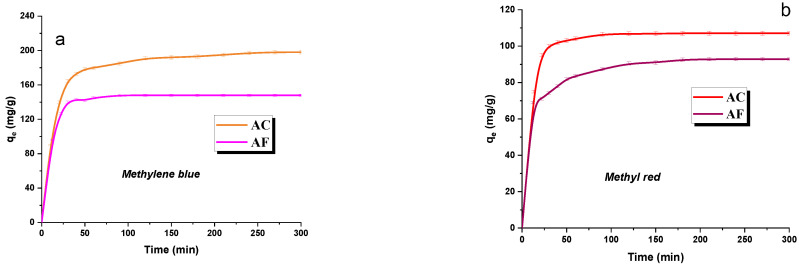
The effect of contact time of adsorbent with adsorbate on the adsorption of methylene blue (**a**)/methyl red (**b**) on activated carbon samples.

Table 5 presents the parameters of the four models: Freudlich, Langmuir, Temin, and Dubinin–Radushkevich. As follows from the values of R^2^, the Langmuir model proved to better describe the data obtained for adsorption of a water solution of methylene blue; the value of R^2^ for sample AC was 0.9986, while for sample AF, it was 0.9913. The highest values of the correlation coefficient for the Langmuir equation obtained for AC and AF indicate that this isotherm provides the best description of methylene blue adsorption. Thus, adsorption of methylene blue on samples AC and AF involves the formation of a monolayer on their surfaces, and the maximum sorption capacity of the monolayer, q_max_, is close to the experimentally determined sorption capacity (Figure 4). For the adsorption of methyl red, the highest correlation coefficient values, R^2^ (0.9911 for AC, 0.9917 for AF), were obtained for the Freundlich model.

According to the data presented in Table 5, the coefficient R_L_ obtained for both samples, AC and AF, irrespectively of the dye adsorbed, takes values from the range 0–1 (methylene blue: AC: 0.254–0.384, AF: 0.502–0.729; methyl red: AC: 0.787–0.908, AF: 0.854–0.976), which favorable adsorption [16]. From the parameters determined for the Temkin model, it can be concluded that the adsorption of water solutions of organic dyes on the surface of AC and AF is an exothermal process as B takes values higher than 0 [32]. The energy of adsorption, E, calculated from the Dubinin–Radushkevich isotherm obtained for AC (methylene blue: 1.763 kJ/mol; methyl red: 0.707 kJ/mol) and AF (methylene blue: 1.581 kJ/mol; methyl red: 0.912 kJ/mol) is lower than 8 kJ/mol, which indicates the process of physisorption. Moreover, the values of the q_m_ parameter obtained for the Dubinin–Radushkevich model are lower than the q_max_ determined for the Langmuir model. The value of q_m_ informs about the maximum amount of adsorbed adsorbate by a given adsorbent in the volume of its micropores [32].

### 3.4. Adsorption Kinetics

The knowledge of the effect of contact time of adsorbent with adsorbate on the effectiveness of adsorption permits the determination of the optimum time of shaking off the adsorbent/adsorbate system to reach equilibrium. The data illustrating this effect are collected in Figure 4a,b and indicate the same tendency irrespective of the dye and the type of activated carbon. According to Figure 4, in the initial phase of the process, a rapid increase in the sorption capacity is observed, which can be explained by the availability of a large number of active centers at which the dye molecules can be easily attached to the adsorbent structure. With increasing time of contact, the number of available active sites decreases, and adsorption slows down until the equilibrium state is reached [40]. As follows from Figure 4, the maximum sorption capacity of samples AC and AF, irrespective of the type of dye, was reached in a relatively short time, which is economically attractive.

On the basis of the results obtained in the experiment checking the effect of contact time on the sorption capacities of samples AC and AF, the kinetic parameters of adsorption were calculated assuming the Langergen model (8) (pseudo-first-order) and Ho and McKay model (9) (pseudo-second-order). The parameters are given in Table 6. A higher value of the correlation coefficient R^2^ was obtained for samples AC (methylene blue: 0.9808; methyl red: 0.9991) and AF (methylene blue: 0.991; methyl red: 0.9985), assuming the pseudo-second-order process suggests the chemisorption of the dyes [41]. Interestingly, a very good agreement between the experimental value of q_e,exp_ parameter, and its value calculated for the Ho and McKay model supports the use of the model for the description of the adsorption of water pollutants on adsorbents obtained from residues of supercritical extraction of green tea leaves.

### 3.5. The Effect of pH of Water Solutions of the Dyes

**Figure 5:**
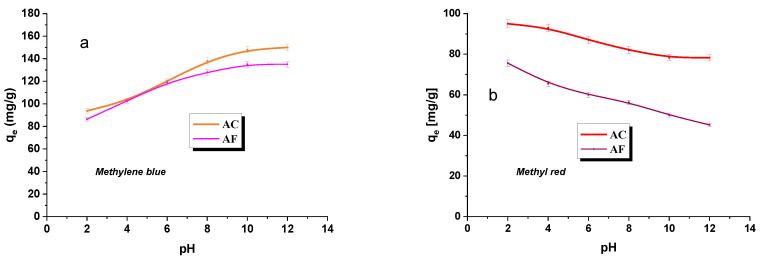
Effect of pH on the adsorption of methylene blue (**a**)/methyl red (**b**) on activated carbon.

The pH value of the water solutions of the dyes is expected to have a significant effect on the effectiveness of their adsorption [42]. The measurements were made for a wide range of pH values, from 2 to 12, and the results are presented in Figure 5a,b. In the process of methylene blue adsorption on samples AC and AF, an increase in the sorption capacity was observed with increasing pH of the adsorbate, while in the process of methyl red adsorption, the relation was quite the reverse. This difference can be explained by the differences in the structure of these dyes: methylene blue is a cationic organic dye, while methyl red is an anionic one [16]. Analysis of the curves presented in Figure 5 also shows that the effect of pH of methylene solution on the effectiveness of its adsorption is greater than that of methyl red. As the adsorbents have acidic as well as basic functional groups on their surface, it can be supposed that the dye molecules are engaged in electrostatic interactions with the surface of the adsorbent. Apart from that, the adsorbents and adsorbates may interact through π-π type interactions [43].

### 3.6. Thermodynamic Study

**Figure 6:**
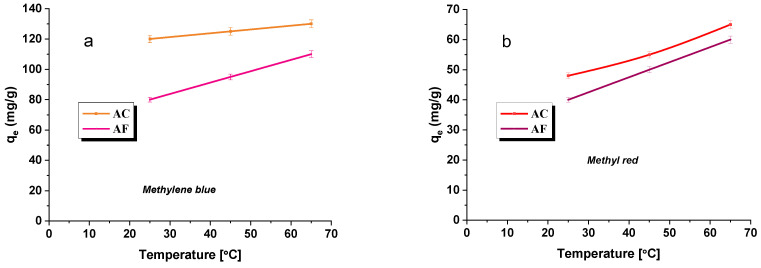
Effect of temperature on the adsorption of methylene blue (**a**)/methyl red (**b**) on activated carbon.

In order to assess the effect of temperature on the sorption capacities of the adsorbents studied, the measurements of absorbance were performed in three temperature variants: at 25, 45, or 65 °C. The results are given in Figure 6, and the thermodynamical parameters calculated from the results are presented in Table 7.

The effect of temperature on the effectiveness of the adsorption of the dye was found to be insignificant. The character of the curves obtained (Figure 6a,b) indicates an increase in the sorption capacities with increasing temperature, but this increase is insignificant, of 20–30 mg of the dye per gram of the adsorbent. That is why we concluded that it is not economically beneficial to run the adsorption at elevated temperatures. On the basis of the performed measurements, it was possible to calculate the following parameters: entropy, enthalpy, and Gibbs free energy, Table 7. The negative values of ΔG indicate the spontaneous character of the process of adsorption, and the degree of its spontaneity increases with increasing temperature. According to the ΔG value, the type of adsorption process can be determined physically or chemically. Generally, for physical adsorption the free energy change ranges from (−20 to 0) kJ/mol, and for chemical adsorption, it ranges between (−80 and −400) kJ/mol. When ∆G values range between −20 and −80 kJ/mol, both physical and chemical adsorption is involved [44,45]. The Gibbs free energy values calculated for the processes of adsorption indicate the physical character of the reaction [46]. Moreover, the positive values of ΔH prove that the methylene blue and methyl red adsorption on the adsorbents studied is the endothermal process [42].

### 3.7. The Effects of Adsorbent Dosage and Agitation Rate

**Figure 7:**
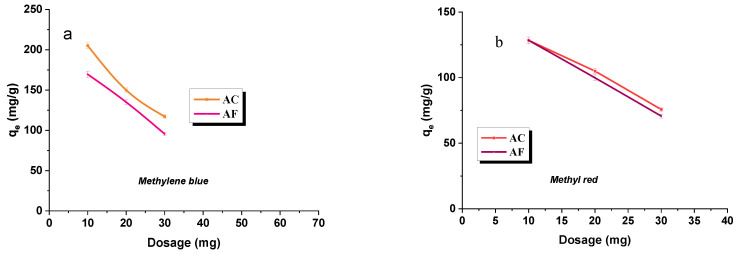
Effect of dosage on the adsorption of methylene blue (**a**)/methyl red (**b**) on activated carbon.

Figure 7a,b presents the data illustrating the effect of adsorbent dosage on the sorption capacities towards methylene blue and methyl red.

**Figure 8:**
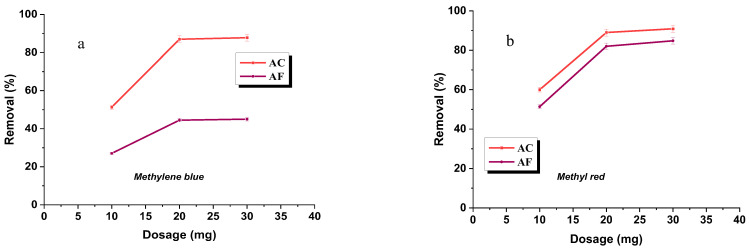
Effect of adsorption efficiency on the adsorption of methylene blue (**a**)/methyl red (**b**) on activated carbon.

As can be concluded from the analysis of these data, with increasing dosage of the adsorbent, the sorption capacity decreases. It can be explained by the decreased ratio of the amount of the dye to the mass unit of the adsorbent, which leads to a decrease in the coefficient describing the use of active sites [47]. It has been found that the efficiency of removal of dyes (Figure 8a,b) from their water solutions on the adsorbents studied increases with increasing dosage of activated carbon, so with the increased surface area and the number of available active sites [48]. No significant changes in adsorption have been noted when applying 20 and 30 mg of the dye [44].

**Figure 9:**
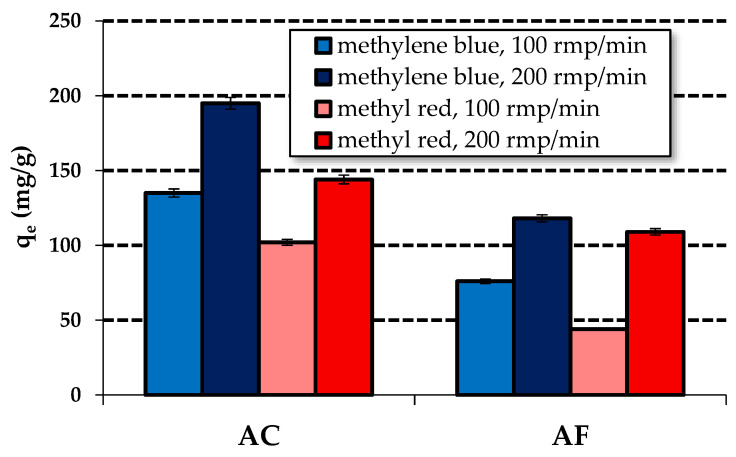
Effect of agitation rate on the adsorption of organic dyes on activated carbon.

Moreover, the effect of the agitation rate on the sorption capacities was also checked, and the results are presented in Figure 9. Analysis of these data has shown that the adsorption of dyes from their water solutions was much more effective at the agitation rate of 200 rmp/min. That is why it was chosen for the experiments.

## 4. Conclusions

The above-described results have confirmed that the method of microwave heating can be successfully applied for obtaining activated carbon-based adsorbents of organic dyes from water solutions. According to the obtained BET results, the adsorbents produced from the residue of supercritical extraction of green tea leaves have a well-developed specific surface area of micro- or micro-mesoporous character. The Langmuir model has better described the experimental results, and the maximum sorption capacity calculated for this model varied from 144.93 to 250.00 mg/g. In order to reach the state of adsorption equilibrium, the process of adsorption of organic dyes from their water solutions should be performed for 5 h, and the kinetics of the process corresponds to the pseudo-second-order model. The negative values of the Gibbs free energy indicate the spontaneous character of the process.

## Figures and Tables

**Table 1:** Characteristics of the organic dyes used.

Organic Dye	Name	Structure	Wavelength [nm]
*methylene blue*	3,7-bis(dimethylamino) -phenothiazin-5-ium	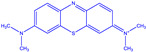	665
*methyl red*	2-[[4(dimethylamino) phenyl]diazenyl]benzoic acid	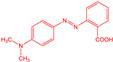	443

**Table 2:** Elemental analysis of agricultural waste materials that can be used as precursors of activated carbons [% wt.].

Precursor	Ash Content	Moisture	Elemental Analysis				References
			C	O	N	H	
*P*	2.1	5.1	58.8	27.2	3.2	10.8	this study
*hops residues*	7.6	-	47.4	38.1	4.8	9.6	[36]
*olive stones*	2.3	8.8	46.5	-	0.4	6.4	[37]
*herb residues*	5.6	4.7	36.1	-	3.5	6.1	[38]
*walnut shells*	1.0	11.2	69.2	25.3	0.3	4.1	[39]

**Table 3:** Textural parameters of the activated carbon samples obtained.

Sample	Surface Area ^1^ [m^2^/g]	Total Pore Volume [cm^3^/g]	Average Pore Diameter [nm]	Micropore Area [m^2^/g]
*AC*	1085	0.64	1.9	1044
*AF*	520	0.62	4.1	360

^1^ Error range between 2–5%.

**Table 4:** Elementary analysis of the activated carbons [% wt.].

Sample	C^daf 1,2^	H^daf^	N^daf^	O^diff 3^
*AC*	83.4	1.6	0.9	14.1
*AF*	88.9	1.4	0.5	9.2

^1^ dry and ash-free state; ^2^ method error ≤0.3%; ^3^ calculated from the difference.

**Table 5:** The isotherm adsorption parameters of methylene blue and methyl red.

Isotherms	Parameters	Methylene Blue Values		Methyl Red Values	
		AC	AF	AC	AF
*Freundlich*	R^2^	0.9966	0.9710	0.9911	0.9917
	K_F_ (mg/g(L/mg)^1/n^)	199.66	93.63	61.67	37.26
	1/n	0.121	0.233	0.353	0.460
*Langmuir*	R^2^	0.9986	0.9913	0.9578	0.9146
	q_m_	250.00	163.93	169.49	144.93
	K_L_ (L/mg)	0.027	0.012	0.003	0.002
	R_L_	0.254–0.384	0.502–0.729	0.787–0.908	0.854–0.976
*Temkin*	R^2^	0.4050	0.7422	0.9809	0.9472
	B	53.81	47.43	43.46	37.73
	A_T_ (L/mg)	12.51	3.30	2.53	1.75
*Dubinin–Radushkevich*	R^2^	0.7608	0.9021	0.8793	0.7953
	q_m_ (mg/g)	228.45	152.47	151.00	108.49
	E (kJ/mol)	1.763	1.581	0.707	0.912

**Table 6:** Kinetic parameters of adsorption of organic dyes.

Isotherms	Parameters	Methylene Blue Values		Methyl Red Values	
		AC	AF	AC	AF
	q_e,exp_ (mg/g)	197.47	147.89	107.14	92.76
*Pseudo-first-order*	R^2^	0.8997	0.9785	0.9670	0.9282
	k_1_ (L/min)	8.52 × 10^−3^	4.44 × 10^−2^	1.65 × 10^−2^	2.09 × 10^−2^
	q_e,cal_ (mg/g)	37.32	39.45	4.54	44.51
*Pseudo-second-order*	R^2^	0.9808	0.9991	0.9991	0.9985
	k_2_ (g/mg × min)	8.17 × 10^−3^	8.63 × 10^−3^	1.63 × 10^−3^	1.58 × 10^−3^
	q_e,cal_ (mg/g)	200.00	149.25	108.69	94.33

**Table 7:** Thermodynamic parameters of the adsorption of organic dyes on the activated carbon adsorbents studied.

Sample	Temperature (K)	∆G (kJ/mol)	∆H (kJ/mol)	∆S (J/mol K)
*AC* *(methylene blue)*	298	−5.32	6.68	40.12
	318	−5.98		
	338	−6.94		
*AF* *(methylene blue)*	298	−4.50	10.15	49.18
	318	−5.47		
	338	−6.47		
*AC* *(methyl red)*	298	−4.77	13.34	60.67
	318	−5.86		
	338	−7.21		
*AF* *(methyl red)*	298	−5.15	19.15	81.44
	318	−6.68		
	338	−8.41		

## Data Availability

Data are contained within the article.

## Supplementary Materials

The following supporting information can be downloaded at: https://www.mdpi.com/article/10.3390/ma15165664/s1, Figure S1: The isotherms obtained in the both ACs.
