# Supplementary figures and images for: Adsorption of Organic Compounds on Adsorbents Obtained with the Use of Microwave Heating
Source: Materials (Basel). 2022 Aug 17;15(16):5664. doi: 10.3390/ma15165664 (PMC9415288; doi:10.3390/ma15165664)

## Supplementary Materials

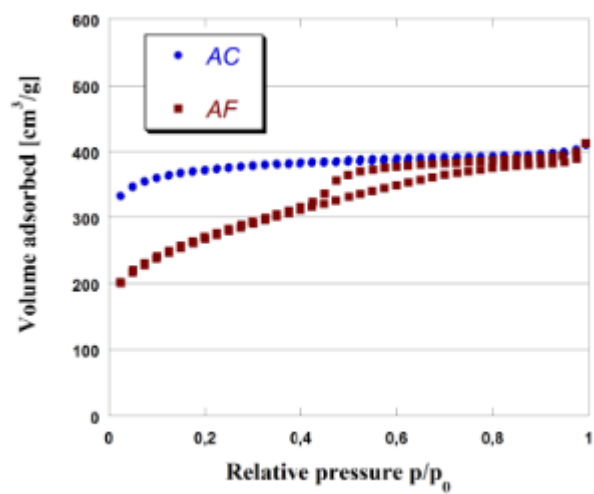

**Figure S1.** The isotherms obtained in the both ACs.

Supplement: Supplementary file 1 [file materials-15-05664-s001.zip › materials-1835509-supplementary.pdf]
